# Association between the melanopsin gene polymorphism OPN4*Ile394Thr and sleep/wake timing in Japanese university students

**DOI:** 10.1186/1880-6805-33-9

**Published:** 2014-05-12

**Authors:** Sang-il Lee, Akiko Hida, Shingo Kitamura, Kazuo Mishima, Shigekazu Higuchi

**Affiliations:** 1 Department of Human Science, Faculty of Design, Kyushu University, 4-9-1 Shiobaru, Minami-ku, Fukuoka 815-8540, Japan; 2 Department of Psychophysiology, National Institute of Mental Health, National Center of Neurology and Psychiatry, 4-1-1 Ogawa-Higashi, Kodaira, Tokyo 187-8553, Japan

**Keywords:** Genotype, Human, Melanopsin gene (*OPN4*), Single nucleotide polymorphism (SNP), Non-image forming responses, Sleep

## Abstract

**Background:**

In our previous studies, we found that the Ile394Thr SNP in the melanopsin gene (*OPN4*) was functionally associated with the pupillary light reflex. This indicates the possibility that OPN4*Ile394Thr is associated with other non-image forming responses. The aim of this study was therefore to determine whether OPN4*Ile394Thr is associated with sleep/wake timing.

**Methods:**

A total of 348 healthy Japanese university students participated in this study. Scalp hair was used to genotype the Ile394Thr SNP of *OPN4*. Sleep habits, including bedtime, wake time and sleep duration, were assessed separately for weekdays and weekends. A total of 328 samples, including 223 samples with TT genotype, 91 with TC genotype and 14 with CC genotype, were used for statistical analysis. No significant difference in age or male/female distribution was found among the three genotype groups.

**Results:**

There was no significant difference in circadian preference among the genotype groups. During weekdays, bedtime, wake time and midpoint of sleep for CC subjects were significantly later than those for TT and TC subjects. However, there was no difference between TT and TC subjects in any of their sleep habits. During weekends, bedtime of CC subjects was significantly later than those of TT and TC subjects, and the midpoint of sleep of CC subjects was significantly later than that of TC subjects.

**Conclusions:**

Our findings demonstrated that OPN4*Ile394Thr is associated with sleep/wake timing. We also found that the sleep/wake timing of subjects with the CC genotype was later than that of subjects with the TT or TC genotype.

## Background

Melanopsin, a photopigment contained in a small subset of retinal ganglion cells, plays an important role in non-image forming (NIF) responses, including circadian photoentrainment [1], melatonin suppression [2], pupillary light reflex [3,4], sleep behavior [5,6] and alertness [7,8], by transmitting photic irradiance information to the brain. Parallel studies using genetic ablation of melanopsin (*Opn4*) in mice [1,9], using a silent-substitution method in humans [4], and using blind subjects [10,11] have demonstrated that the contribution of melanopsin to NIF responses is as important as, or even more important than, that of the classical photoreceptors (rods and cones).

In our previous studies, we found that the Ile394Thr SNP (rs1079610) in the melanopsin gene (*OPN4*) was functionally associated with pupillary light reflex (PLR) and that subjects with different genotypes of Ile394Thr SNP showed different degrees of responsiveness to light [12,13]. Thus, OPN4*Ile394Thr might be a factor involved in inter-individual differences in other NIF responses depending on light, such as circadian phase-shifting, but this remains unclear.

The endogenous circadian clock in mammals is a self-sustained oscillation with a period of about 24 hours. In fact, the circadian clock runs free without entrainment (synchronization) to environmental signals, especially the light/dark cycle in a day. As mentioned above, parallel studies have demonstrated an important role of melanopsin in circadian entrainment [1,5,11,14]. Melanopsin-containing retinal ganglion cells detect irradiance information and transmit the photic signal to the suprachiasmatic nucleus, the circadian pacemaker, located in the hypothalamus [15].

An individual circadian phase shows a light intensity-dependent manner and can be advanced or delayed depending on exposure timing to light. Notably, Zeitzer *et al*. [16] found that phase-delaying effects of light were increased in humans during early night and that the delayed phase appeared with exposure to not only bright light but also low irradiance light. It is therefore possible that the different degrees of responsiveness to light among OPN4*Ile394Thr genotypes may influence circadian phase.

Sleep/wake timing in humans is thought to reflect circadian phase [17,18] and to be correlated with dim light melatonin onset (DLMO) phase, which has been used to estimate an individual’s circadian phase [19,20]. Aoki *et al*. [21] found that the magnitude of light-induced melatonin suppression in patients with DSPS (delayed sleep phase syndrome) was greater than that in normal subjects. This indicates the possibility that inter-individual differences in circadian phase are associated with photoreceptor light sensitivity.

Taken together, we hypothesized that the effect of Ile394Thr SNP on circadian phase is reflected in sleep/wake timing. Hence, the aim of this study was to determine whether OPN4*Ile394Thr is associated with sleep/wake timing.

## Methods

### Subjects

A total of 348 healthy Japanese university students (mean age: 20.9 years; SD: 2.2) with common color vision (Ishihara color-blindness test) participated in this study. Exclusion criteria included medication or drug consumption and shift work. All participants were enrolled with written consent of each participant, and the study was approved by the Ethical Committee of Kyushu University and the Ethics committee of the National Center of Neurology and Psychiatry. There was no significant difference in age or male/female distribution among the three genotype groups. Table 1 shows the demographical characteristics of the subjects.

**Table 1:** Demographical characteristics of each genotype group

**Genotype**	**TT**	**TC**	**CC**	***P-*** **value**
n	223	91	14	
Sex (M:F)	122:101	49:42	4:10	0.163 (*χ*^2^)
Age (years ± SD)	21.0 ± 2.3	20.8 ± 2.0	21.3 ± 1.5	0.667

### Investigation of circadian preference and sleep-wake timing

So-called morningness-eveningness, namely circadian preference, is an individual characteristic and shows a strong correlation with sleep/wake timing [22]. A Japanese version of the Morningness-Eveningness Questionnaire (MEQ) [23] was used to evaluate the effect of individual circadian preference on sleep/wake timing. In addition, sleep habits (bedtime, wake time and sleep duration) were assessed separately for weekdays and weekends, because individual sleep/wake times have been shown to differ greatly between weekdays and weekends [24]. Besides sleep/wake timing, midpoint of sleep has also been used to estimate an individual’s circadian phase, and it has been shown to have a strong correlation with DLMO phase [25,26]. The midpoint of sleep was calculated on the basis of self-reported bedtime and wake time.

### Genotyping

Genomic DNA samples were extracted from a scalp hair using an FM Kit (Wako Pure Chemical Industries, Ltd., Osaka, Japan), and Ile394Thr SNP was genotyped in all participants by using TaqMan SNP genotyping assays (Applied Biosystems, Foster City, California, USA). The genotype groups were classified as TT, TC and CC, and the numbers of subjects in those groups were 232, 94 and 14, respectively (eight being undetermined). Genotype frequency of OPN4*Ile394Thr was consistent with the Hardy-Weinberg equilibrium (*χ*^2^ = 2.12, ns), and the T and C allele frequencies of Ile394Thr SNP were 82.1% and 17.9%, respectively.

### Statistical analysis

Subjects who did not complete the self-assessment questionnaire were excluded. After exclusion, a total of 328 samples, including 223 samples with TT genotype (122 men and 101 women; 21.0 ± 2.3 years old), 91 samples with TC genotype (49 men and 42 women, 20.8 ± 2.0 years old) and 14 samples with CC genotype (4 men and 10 women, 21.3 ± 1.5 years old), were used for statistical analysis (Table 1).

To evaluate the differences among the genotype groups for dependent variables, including sleep habits and circadian preference, we used one-way multivariate ANOVA (IBM^©^ SPSS^©^ version 21, New York, USA) with the genotypes as independent variables. Tukey (honestly significant difference) HSD *post hoc* tests were carried out when the interaction between genotypes and each dependent variable was significant. *P* < 0.05 was considered to be statistically significant.

## Results

The MEQ mean scores and standard deviations were 48.4 ± 7.4 in the TT subjects, 48.6 ± 7.3 in the TC subjects and 44.8 ± 6.6 in the CC subjects (Table 1). ANOVA showed no significant difference among OPN4*Ile394Thr genotypes.

**Figure 1:**
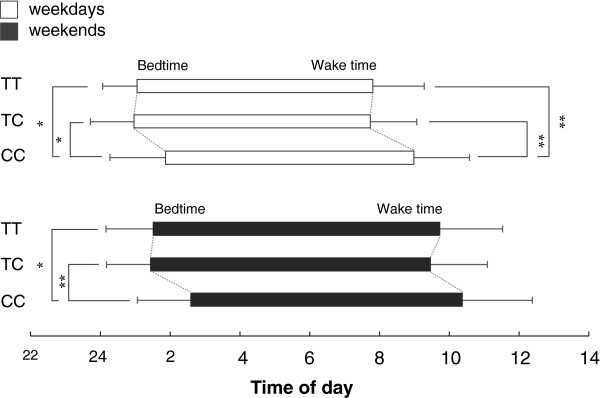
**Comparison of bedtime and wake time (mean + SD) among TT (n = 223), TC (n = 91) and CC (n = 14) subjects during weekdays (white bars) and weekends (black bars).** During weekdays, both the bedtime and wake time of CC subjects were significantly later than those of TT and TC subjects. During weekends, the results for bedtime were consistent with those during weekdays, but there were no significant differences among genotype groups in wake time. *P < 0.05, **P < 0.01.

**Figure 2:**
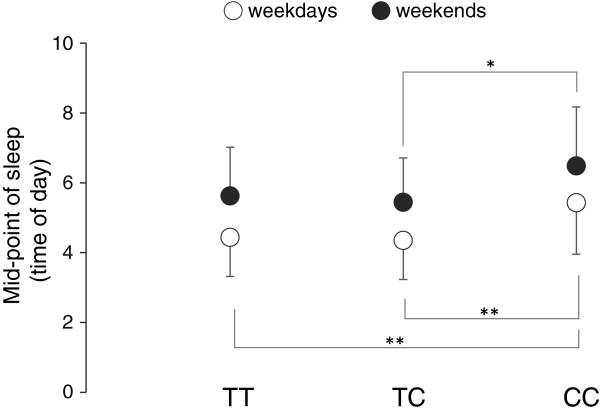
**Comparison of midpoint of sleep (mean + SD) among TT (n = 223), TC (n = 91) and CC (n = 14) subjects during weekdays (white circles) and weekends (black circles).** During weekdays, the midpoint of sleep of CC subjects was significantly later than those of TT and TC subjects. During weekends, there was a significant difference in midpoint of sleep only between TC and CC subjects. *P < 0.05, **P < 0.01.

ANOVA for the data during weekdays showed main effects of genotype on bedtime (F = 7.058; *P* < 0.01), wake time (F = 3.353; *P* < 0.05) and midpoint of sleep (F = 5.622; *P* < 0.01). No significant effect of genotype on sleep duration was found. In the sleep habits during weekends, main effects of genotype were found on bedtime (F = 5.624; *P* < 0.01) and midpoint of sleep (F = 3.964; *P* < 0.05) but not on wake time or sleep duration (Table 2).Figure 1 shows bedtime and wake time of each genotype group both on weekdays and weekends. During weekdays, CC subjects reported significantly later bedtimes than those of TT subjects and TC subjects as well as later wake times. During weekends, CC subjects showed later bedtime than those for TT subjects and TC subjects, while there was no significant difference among the genotype groups in wake time. No significant difference was found between TT and TC subjects in any of the sleep habits.The midpoint of sleep for CC subjects was significantly later than those for the TT subjects and TC subjects on weekdays, but no significant difference was found between TT subjects and TC subjects. The midpoint of sleep for the CC subjects was later than that for the TC subjects on weekends, but no significant difference was found between TT subjects and CC subjects or between TC subjects and TT subjects (Figure 2).

**Table 2:** Morningness-Eveningness Questionnaire (MEQ) score and sleep habits of each genotype group

**Genotype**	**TT (n = 223)**	**TC (n = 91)**	**CC (n = 14)**
MEQ score	48.4 ± 7.4	48.6 ± 7.3	44.8 ± 6.6
Weekdays			
Bedtime	1:03 ± 0:59	0:58 ± 1:14	1:52 ± 1:36
Wake time	7:49 ± 1:28	7:44 ± 1:20	8:59 ± 1:35
Mid-sleep	4:26 ± 1:06	8:17 ± 1:07	5:26 ± 1:29
Sleep duration	6:16 ± 1:02	6:25 ± 1:15	6:58 ± 1:20
Weekends			
Bedtime	1:31 ± 1:21	1:26 ± 1:15	2:35 ± 1:31
Wake time	9:44 ± 1:47	9:26 ± 1:38	10:23 ± 2:00
Mid-sleep	5:37 ± 1:24	5:26 ± 1:17	6:29 ± 1:41
Sleep duration	8:08 ± 1:26	8:03 ± 1:41	7:45 ± 1:27
Values indicate means ± SD.			

## Discussion

We attempted to determine the association between OPN4*Ile394Thr and sleep habits. We found that subjects with the CC genotype had later sleep/wake timing and later midpoint of sleep than those for subjects with the TT or TC genotype, indicating that OPN4*Ile394Thr is associated with sleep/wake timing. On the other hand, the circadian preferences identified by MEQ scores showed similar trends among the genotype groups, indicating that the sleep habits of subjects with each genotype were not biased by individual preference.

Light is a critical environmental cue for circadian entrainment and circadian phase can be advanced or delayed depending on light intensity and exposure timing. Exposure to light at night results in phase delay. In support of this, it has been reported that exposure of human subjects to ordinary room light with low irradiance (approximately 300 lux) during the night caused delayed circadian phase [16,27], melatonin suppression [28], and alertness [29]. All of the subjects who participated in the present study were university students and had late bedtimes (mean bedtime: 1:15 AM), which means, although we did not assess light conditions for the subjects in daily life, it can be assumed that the subjects were exposed to indoor light for a long duration during the night. Thus, it is possible that the findings in this study were due to the effect of light exposure at night.

In our previous study, we found that pupilloconstriction of TC and CC subjects or a combined group (TC + CC) was greater than that of TT subjects, suggesting that the genotype with C allele is highly responsive to light [12,13]. Given that the phase-delaying response to light follows a logistic dose–response curve [16], the delayed sleep/wake timing of CC subjects might be a consequence of the high responsiveness to light.

Unlike on weekends, it is difficult for an individual circadian phase, which is strongly affected by social constraints, especially wake time, to be reflected in the sleep/wake cycle on weekdays. Despite this, delayed sleep phase of the CC subjects was observed more clearly during weekdays than during weekends. Although university students are under a time constraint on weekdays, the level of the constraint is weak. In addition, sleep is generated by the interaction between circadian and homeostatic mechanisms, and the latter mechanism is thought to be an increase in sleep pressure during wakefulness and dissipation during subsequent sleep. Particularly in adolescents, sleep debt is likely to accumulate during weekdays and therefore lead to oversleep as a compensation for sleep loss during weekends. Thus, the disappearance of a statistical difference among genotypes in wake time and midpoint of sleep during weekends might be due to homeostatic sleep regulation.

However, the relationship between OPN4*Ile394Thr and sleep habits in this study was not consistent with the results for the relationship between OPN4*Ile394Thr and PLR in our previous study: sleep/wake timing of the CC subjects was significantly delayed compared to that of the TT and TC subjects, whereas no difference was found between the TC and CC subjects in PLR. It is not clear what caused this, but it indicates that the previous results for PLR cannot sufficiently explain the sleep phase difference among OPN4*Ile394Thr genotypes. Compared to PLR, a more complicated mechanism and more factors are involved in sleep. For instance, inter-individual differences in endogenous circadian rhythm, circadian phase and sleep timing have been reported [30,31]. Furthermore, contribution of the *CLOCK* gene to circadian phenotypes, particularly sleep timing, has been reported [32,33].

Furthermore, melanopsin has a characteristic spectral sensitivity λ_max_ around 480 nm. The effect of light with a high-color temperature (that is, blue-enriched) on melatonin suppression or sleep has been investigated [34-36]. Also, in our previous study, we found that the difference between OPN4*Ile394Thr genotypes in PLR depends on light wavelength: greater differences were observed with a short wavelength light [13]. Therefore, in future work, those factors should be evaluated to validate the findings in this study.

The sample size for CC genotype was small (n = 14) compared to TT and TC subjects. As International HapMap Project reported, the frequency of CC genotype is relatively low not only in Japanese in Tokyo (2.3%) but also in other ethnic groups; for example, 13.3% of Han Chinese in Beijing and 12.3% of European ancestry in Utah state. To enhance confidence in our findings, larger samples will be required.

### Perspective

According to the database of International HapMap Project, C allele frequency of Ile394Thr SNP in CEU (European ancestry in Utah state, 34.2%) is larger than that in JPT (Japanese in Tokyo, 17.0%) and that in YRI (Yoruba in Nigeria, 14.2%). It was also found that pupillary light response in CC genotype is larger in the European population [37]. These findings mean that the proportion of people with high sensitivity to light might be relatively large in the European population. It is thought that light skin color in the European population results from genetic adaptation to a short duration of sunlight in a high latitude area. Another example of the health risk of a short duration of sunlight is seasonal affective disorder (SAD), which is involved in the mechanism of melanopsin-containing non-visual response to light. Although it has been reported that melanopsin gene polymorphism is associated with prevalence of SAD [38], it is unknown whether natural selection has driven it or not. Further study based on population genetics, such as a statistical approach to estimate the degree of population differentiation (Wright’s Fst) [39] and to measure linkage disequilibrium as evidence of selective sweep [40], is needed.

Although we found an association between OPN4*Ile394Thr and sleep timing in this study, an association was not found in another study that was conducted in the USA using middle-aged European subjects [41]. This inconsistency suggests that associations between genotype and phenotypic variations are not simple and that these relations are modulated by environment and age. In the present study, the association between *OPN4* polymorphism and sleep timing was thought to have been due to the effect of light at night on the circadian phase. In Japan, most people are likely to use a fluorescent lamp at home and some tend to use high-color temperature light, which has an impact on sleep and circadian rhythm. In addition, our subjects were university students, who have been reported to have a tendency to delay sleep timing due to weak social *zeitgebers*[42]. These cultural and environmental factors in Japanese university students might strengthen the association between *OPN4* polymorphism and sleep timing. In physiological anthropology, functional connections and biological significance between genotypic and phenotypic variations should be clarified in terms of interaction of culture and living environments in a targeted population.

## Conclusion

Our findings demonstrated that OPN4*Ile394Thr is associated with sleep/wake timing. We also found that the sleep/wake timing of subjects with the CC genotype was later than that of subjects with the TT or TC genotype.

## Abbreviations

DLMO: dim light melatonin onset; DSPS: delayed sleep phase syndrome; MEQ: Morningness-Eveningness Questionnaire; NIF: non-image forming; PLR: pupillary light reflex; SAD: seasonal affective disorder; SNP: single nucleotide polymorphism.

## Competing interests

The authors declare that they have no competing interests.

## Authors’ contributions

SL collected experimental data, performed statistical analysis and wrote the manuscript. SL and SH participated in the design of the study. SL and AH carried out the molecular genetic analysis. SK, AH and KM revised the manuscript. SH supervised the study and helped to draft the manuscript. All authors read and approved the final manuscript.

## References

[B1] Panda S Sato TK Castrucci AM Rollag MD DeGrip WJ Hogenesch JB Provencio I Kay SA Melanopsin (Opn4) requirement for normal light-induced circadian phase shifting Science 2002 298 2213 2216 10.1126/science.1076848 12481141

[B2] Brainard GC Hanifin JP Rollag MD Greeson J Byrne B Glickman G Gerner E Sanford B Human melatonin regulation is not mediated by the three cone photopic visual system J Clin Endocrinol Metab 2001 86 433 436 10.1210/jcem.86.1.7277 11232036

[B3] Gamlin PD McDougal DH Pokorny J Smith VC Yau KW Dacey DM Human and macaque pupil responses driven by melanopsin-containing retinal ganglion cells Vision Res 2007 47 946 954 10.1016/j.visres.2006.12.015 17320141 PMC1945238

[B4] Tsujimura S Ukai K Ohama D Nuruki A Yunokuchi K Contribution of human melanopsin retinal ganglion cells to steady-state pupil responses Proc Biol Sci 2010 277 2485 2492 10.1098/rspb.2010.0330 20375057 PMC2894926

[B5] Lupi D Oster H Thompson S Foster RG The acute light-induction of sleep is mediated by OPN4-based photoreception Nat Neurosci 2008 11 1068 1073 10.1038/nn.2179 19160505

[B6] Tsai JW Hannibal J Hagiwara G Colas D Ruppert E Ruby NF Heller HC Franken P Bourgin P Melanopsin as a sleep modulator: circadian gating of the direct effects of light on sleep and altered sleep homeostasis in Opn4(−/−) mice Plos Biol 2009 7 e1000125 10.1371/journal.pbio.1000125 19513122 PMC2688840

[B7] Cajochen C Munch M Kobialka S Krauchi K Steiner R Oelhafen P Orgul S Wirz-Justice A High sensitivity of human melatonin, alertness, thermoregulation, and heart rate to short wavelength light J Clin Endocrinol Metab 2005 90 1311 1316 10.1210/jc.2004-0957 15585546

[B8] An M Huang J Shimomura Y Katsuura T Time-of-day-dependent effects of monochromatic light exposure on human cognitive function J Physiol Anthropol 2009 28 217 223 10.2114/jpa2.28.217 19823003

[B9] Lucas RJ Hattar S Takao M Berson DM Foster RG Yau KW Diminished pupillary light reflex at high irradiances in melanopsin-knockout mice Science 2003 299 245 247 10.1126/science.1077293 12522249

[B10] Gooley JJ Mien IH St Hilaire MA Yeo SC Chua ECP van Reen E Hanley CJ Hull JT Czeisler CA Lockley SW Melanopsin and rod-cone photoreceptors play different roles in mediating pupillary light responses during exposure to continuous light in humans J Neurosci 2012 32 14242 14253 10.1523/JNEUROSCI.1321-12.2012 23055493 PMC3515688

[B11] Zaidi FH Hull JT Peirson SN Wulff K Aeschbach D Gooley JJ Brainard GC Gregory-Evans K Rizzo JF Czeisler CA Foster RG Moseley MJ Lockley SW Short-wavelength light sensitivity of circadian, pupillary, and visual awareness in humans lacking an outer retina Curr Biol 2007 17 2122 2128 10.1016/j.cub.2007.11.034 18082405 PMC2151130

[B12] Higuchi S Hida A Tsujimura S Mishima K Yasukouchi A Lee SI Kinjyo Y Miyahira M Melanopsin gene polymorphism *I394T* is associated with pupillary light responses in a dose-dependent manner Plos One 2013 8 e60310 10.1371/journal.pone.0060310 23555953 PMC3610661

[B13] Lee SI Hida A Tsujimura S Morita T Mishima K Higuchi S Association between melanopsin gene polymorphism (*I394T*) and pupillary light reflex is dependent on light wavelength J Physiol Anthropol 2013 32 16 10.1186/1880-6805-32-16 24119231 PMC4015917

[B14] Altimus CM Guler AD Villa KL McNeill DS Legates TA Hattar S Rods-cones and melanopsin detect light and dark to modulate sleep independent of image formation Proc Natl Acad Sci U S A 2008 105 19998 20003 10.1073/pnas.0808312105 19060203 PMC2596746

[B15] Hattar S Kumar M Park A Tong P Tung J Yau KW Berson DM Central projections of melanopsin-expressing retinal ganglion cells in the mouse J Comp Neurol 2006 497 326 349 10.1002/cne.20970 16736474 PMC2885916

[B16] Zeitzer JM Dijk DJ Kronauer R Brown E Czeisler C Sensitivity of the human circadian pacemaker to nocturnal light: melatonin phase resetting and suppression J Physiol 2000 526 Pt 3 695 702 10922269 10.1111/j.1469-7793.2000.00695.x PMC2270041

[B17] Borbely AA A two process model of sleep regulation Human Neurobiol 1982 1 195 204 7185792

[B18] Dijk DJ Czeisler CA Paradoxical timing of the circadian rhythm of sleep propensity serves to consolidate sleep and wakefulness in humans Neurosci Lett 1994 166 63 68 10.1016/0304-3940(94)90841-9 8190360

[B19] Burgess HJ Eastman CI The dim light melatonin onset following fixed and free sleep schedules J Sleep Res 2005 14 229 237 10.1111/j.1365-2869.2005.00470.x 16120097 PMC3841975

[B20] Martin SK Eastman CI Sleep logs of young adults with self-selected sleep times predict the dim light melatonin onset Chronobiol Int 2002 19 695 707 10.1081/CBI-120006080 12182497

[B21] Aoki H Ozeki Y Yamada N Hypersensitivity of melatonin suppression in response to light in patients with delayed sleep phase syndrome Chronobio Int 2001 18 263 271 10.1081/CBI-100103190 11379666

[B22] Mongrain V Lavoie S Selmaoui B Paquet J Dumont M Phase relationships between sleep-wake cycle and underlying circadian rhythms in Morningness-Eveningness J Biol Rhythms 2004 19 248 257 10.1177/0748730404264365 15155011

[B23] Horne JA Ostberg O A self-assessment questionnaire to determine morningness-eveningness in human circadian rhythms Int J Chronobiol 1976 4 97 110 1027738

[B24] Roenneberg T Wirz-Justice A Merrow M Life between clocks: daily temporal patterns of human chronotypes J Biol Rhythms 2003 18 80 90 10.1177/0748730402239679 12568247

[B25] Crowley SJ Acebo C Fallone G Carskadon MA Estimating dim light melatonin onset (DLMO) phase in adolescents using summer or school-year sleep/wake schedules Sleep 2006 29 1632 1641 17252895 10.1093/sleep/29.12.1632

[B26] Terman JS Terman M Lo ES Cooper TB Circadian time of morning light administration and therapeutic response in winter depression Arch Gen Psychiatry 2001 58 69 75 10.1001/archpsyc.58.1.69 11146760

[B27] Burgess HJ Evening ambient light exposure can reduce circadian phase advances to morning light independent of sleep deprivation J Sleep Res 2013 22 83 88 10.1111/j.1365-2869.2012.01042.x 22889464 PMC3731138

[B28] Gooley JJ Chamberlain K Smith KA Khalsa SB Rajaratnam SM Van Reen E Zeitzer JM Czeisler CA Lockley SW Exposure to room light before bedtime suppresses melatonin onset and shortens melatonin duration in humans J Clin Endocrinol Metab 2011 96 E463 E472 10.1210/jc.2010-2098 21193540 PMC3047226

[B29] Cajochen C Zeitzer JM Czeisler CA Dijk DJ Dose–response relationship for light intensity and ocular and electroencephalographic correlates of human alertness Behav Brain Res 2000 115 75 83 10996410 10.1016/s0166-4328(00)00236-9

[B30] Duffy JF Rimmer DW Czeisler CA Association of intrinsic circadian period with morningness-eveningness, usual wake time, and circadian phase Behav Neurosci 2001 115 895 899 11508728 10.1037//0735-7044.115.4.895

[B31] Hida A Kitamura S Ohsawa Y Enomoto M Katayose Y Motomura Y Moriguchi Y Nozaki K Watanabe M Aritake S Higuchi S Kato M Kamei Y Yamazaki S Goto Y Ikeda M Mishima K *In vitro* circadian period is associated with circadian/sleep preference Sci Rep 2013 2074 3 10.1038/srep02074 PMC3691610 23797865

[B32] Ebisawa T Uchiyama M Kajimura N Mishima K Kamei Y Katoh M Watanabe T Sekimoto M Shibui K Kim K Kudo Y Ozeki Y Sugishita M Toyoshima R Inoue Y Yamada N Nagase T Ozaki N Ohara O Ishida N Okawa M Takahashi K Yamauchi T Association of structural polymorphisms in the human *period3* gene with delayed sleep phase syndrome EMBO Rep 2001 2 342 346 10.1093/embo-reports/kve070 11306557 PMC1083867

[B33] Mishima K Tozawa T Satoh K Saitoh H Mishima Y The 3111 T/C polymorphism of *hClock* is associated with evening preference and delayed sleep timing in a Japanese population sample Am J Med Genet B Neuropsychiatr Genet 2005 133B 101 104 10.1002/ajmg.b.30110 15578592

[B34] Chellappa SL Steiner R Blattner P Oelhafen P Gotz T Cajochen C Non-visual effects of light on melatonin, alertness and cognitive performance: can blue-enriched light keep us alert? Plos One 2011 6 e16429 10.1371/journal.pone.0016429 21298068 PMC3027693

[B35] Kozaki T Kitamura S Higashihara Y Ishibashi K Noguchi H Yasukouchi A Effect of color temperature of light sources on slow-wave sleep J Physiol Anthropol Appl Human Sci 2005 24 183 186 10.2114/jpa.24.183 15840951

[B36] Santhi N Thorne HC van der Veen DR Johnsen S Mills SL Hommes V Schlangen LJ Archer SN Dijk DJ The spectral composition of evening light and individual differences in the suppression of melatonin and delay of sleep in humans J Pineal Res 2012 53 47 59 10.1111/j.1600-079X.2011.00970.x 22017511

[B37] Roecklein K Wong P Ernecoff N Miller M Donofry S Kamarck M Wood-Vasey WM Franzen P The post illumination pupil response is reduced in seasonal affective disorder Psychiatry Res 2013 210 150 158 10.1016/j.psychres.2013.05.023 23809464 PMC3795919

[B38] Roecklein KA Rohan KJ Duncan WC Rollag MD Rosenthal NE Lipsky RH Provencio I A missense variant (*P10L*) of the melanopsin (*OPN4*) gene in seasonal affective disorder J Affect Disord 2009 114 279 285 10.1016/j.jad.2008.08.005 18804284 PMC2647333

[B39] Wright S Evolution and the genetics of populations. Vol 2: The theory of gene frequencies 1969 Chicago: University of Chicago press

[B40] The International HapMap Consortium A haplotype map of the human genome Nature 2005 437 1299 1320 10.1038/nature04226 16255080 PMC1880871

[B41] Roecklein KA Wong PM Franzen PL Hasler BP Wood-Vasey WM Nimgaonkar VL Miller MA Kepreos KM Ferrell RE Manuck SB Melanopsin gene variations interact with season to predict sleep onset and chronotype Chronobiol Int 2012 29 1036 1047 10.3109/07420528.2012.706766 22881342 PMC3724237

[B42] Urner M Tornic J Bloch KE Sleep patterns in high school and university students: a longitudinal study Chronobiol Int 2009 26 1222 1234 10.3109/07420520903244600 19731114

